# Essential Oils and Sustainability: In Vitro Bioactivity Screening of *Myristica fragrans* Houtt. Post-Distillation By-Products

**DOI:** 10.3390/plants12091741

**Published:** 2023-04-23

**Authors:** Adriana Trifan, Gokhan Zengin, Izabela Korona-Glowniak, Krystyna Skalicka-Woźniak, Simon Vlad Luca

**Affiliations:** 1 Department of Pharmacognosy-Phytotherapy, Faculty of Pharmacy, “Grigore T. Popa” University of Medicine and Pharmacy Iasi, 700115 Iasi, Romania; 2 Physiology and Biochemistry Research Laboratory, Department of Biology, Science Faculty, Selcuk University, Konya 42130, Turkey; 3 Department of Pharmaceutical Microbiology, Faculty of Pharmacy, Medical University of Lublin, 20-093 Lublin, Poland; iza.glowniak@umlub.pl; 4 Department of Natural Products Chemistry, Medical University of Lublin, 20-093 Lublin, Poland; kskalicka@pharmacognosy.org; 5 Biothermodynamics, TUM School of Life and Food Sciences, Technical University of Munich, 85354 Freising, Germany; vlad.luca@tum.de

**Keywords:** nutmeg, spices, wastes, essential oils, spent material, LC-HRMS/MS

## Abstract

The essential oil of *Myristica fragrans* Hutt. (nutmeg) is an important commodity used as a flavoring agent in the food, pharmaceutical, and cosmetic fields. Hydrodistillation is chiefly employed at the industrial scale for nutmeg essential oil isolation, but such a technique generates large quantities of post-distillation by-products (e.g., spent plant material and residual distillation water). Therefore, our work aimed to propose a novel strategy for the valorization of nutmeg wastes, with beneficial economic and ecological advantages. Thus, the current study assessed the phytochemical (GC-MS, LC-HRMS/MS) and biological (antioxidant, enzyme inhibitory, antimicrobial) profile of nutmeg crude materials (essential oil and total extract) and post-distillation by-products (residual water and spent material extract). Identified in these were 43 volatile compounds, with sabinene (21.71%), *α*-pinene (15.81%), myristicin (13.39%), and *β*-pinene (12.70%) as the main constituents. LC-HRMS/MS analysis of the nutmeg extracts noted fifteen metabolites (e.g., organic acids, flavonoids, phenolic acids, lignans, and diarylnonanoids). Among the investigated nutmeg samples, the spent material extract was highlighted as an important source of bioactive compounds, with a total phenolic and flavonoid content of 63.31 ± 0.72 mg GAE/g and 8.31 ± 0.06 mg RE/g, respectively. Moreover, it showed prominent radical-scavenging and metal-reducing properties and significantly inhibited butyrylcholinesterase (4.78 ± 0.03 mg GALAE/g). Further, the spent material extract displayed strong antimicrobial effects against *Streptococcus pneumoniae*, *Micrococcus luteus*, and *Bacillus cereus* (minimum inhibitory concentrations of 62.5 mg/L). Overall, our study brings evidence on the health-promoting (antioxidant, anti-enzymatic, antimicrobial) potential of nutmeg post-distillation by-products with future reference to their valorization in the pharmaceutical, cosmeceutical, and food industries.

## 1. Introduction

*Myristica fragrans* Houtt. (nutmeg) is an evergreen tree indigenous to the tropical forests of the Maluku Islands, currently being distributed and cultivated in Indonesia and Caribbean Grenada Islands, but also in India, Sri Lanka, Mauritius, South Africa, the majority of African countries, and the United States of America [1]. Nutmeg seeds have a specific spicy fragrance and a fairly sweet taste and therefore are commonly added as a spice for flavoring foods and beverages (e.g., meat, fish, sausages, soups, vegetables, cakes, biscuits, buns, pies, syrups, eggnog, and puddings) [2]. Besides its culinary applications, nutmeg has been used as a traditional remedy for alleviating skin infections, rheumatism, and gastrointestinal, central nervous system, and kidney disorders [3]. Nutmeg seeds are a rich source of essential oil (up to 16%), fixed oil (“nutmeg butter”, ~40%), saponins, and various phenolic compounds (lignans and neolignans, phenolic acids, tannins, and flavonoids). Literature data have revealed that nutmeg metabolites possess a plethora of biological activities such as antimicrobial, analgesic, anti-inflammatory, antidepressant, memory-enhancing, anticancer, anti-diabetic, anti-obesity, antioxidant, and hepatoprotective effects that might support its use in traditional medicine [2,4].

In particular, the essential oil derived from nutmeg seeds is an important commodity, being widespread as a flavoring agent in processed foods and drinks and thus replacing ground nutmeg. The United States Food and Drug Administration (FDA) classified nutmeg essential oil as generally recognized as safe (GRAS) and much interest was given to its use as a biopreservative [5]. Hydrodistillation is chiefly used at an industrial scale for nutmeg essential oil extraction, as it is a simple method that allows for a short processing time for large quantities of biomass with reduced labor costs [6]. Still, such a technique generates large amounts of by-products such as spent plant material (solid waste, up to 94% of the raw material) and residual distillation waters (wastewaters or leachates) [7]. Since this waste is disposed of in the environment without any further processing, novel strategies for its recycling are in high demand. Therefore, environmentally friendly reuse of solid wastes as compost in agriculture, alternative energy sources, or antioxidant adjuvants in biodiesel production has been proposed [8,9,10,11]. In addition, recent studies showed that post-distillation by-products are valuable sources of bioactive compounds with potential uses in the pharmaceutical, food, and cosmeceutical fields [12,13,14,15,16,17].

However, scientific data on the recovery and re-utilization of nutmeg post-distillation by-products are lacking. A literature survey revealed that only one study suggested the conversion of nutmeg solid wastes into biomass briquettes as energy sources for industry production activities [7]. Therefore, innovative means of assigning value to nutmeg wastes could have a significant economic and ecological impact. In this respect, our study aimed to comparatively assess the phytochemical and biological profile of nutmeg raw materials (essential oil, total extract) and post-distillation by-products (spent material and residual water extracts). The essential oil and extracts analysis was undertaken by gas chromatography coupled with mass spectrometry (GC-MS) and liquid chromatography coupled with high-resolution tandem mass spectrometry (LC-HRMS/MS), respectively. Further, the bioactivity of nutmeg samples was investigated by in vitro antioxidant (radical scavenging, metal-chelating, and metal-reducing), anti-enzymatic (anti-acetylcholinesterase, anti-butyrylcholinesterase, anti-amylase, anti-glucosidase, and anti-tyrosinase), and antimicrobial (against Gram-positive and Gram-negative bacteria and yeast strains) assays.

## 2. Results and Discussion

### 2.1. GC-MS Analysis of Nutmeg Essential Oil

Nutmeg essential oil (NEO) was obtained by hydrodistillation following the methodology detailed in Section 3.2.1. and was further characterized by GC-MS analysis (Table 1).

A total of 43 volatile constituents were identified in the nutmeg essential oil, representing approximately 99% of the total compounds (as determined from the GC-MS chromatograms). The identified compounds belong to five different groups, with a prevalence of hydrocarbon monoterpenes (68.50%), aromatic compounds (23.31%), and oxygenated monoterpenes (6.41%). The major constituents found in the nutmeg essential oil were sabinene (21.71%), α-pinene (15.81%), myristicin (13.39%), and β-pinene (12.70%). Our results are in agreement with the chemical composition of nutmeg essential oil reported by previous studies in terms of marker compounds. For instance, Dupuy et al. [18] analyzed fourteen nutmeg essential oil samples of different origins and reported similar mean values for sabinene (25.56%), *α*-pinene (16.73%), *β*-pinene (12.36%), and myristicin (8.13%), respectively. Additionally, the main constituents from an Iranian sample of nutmeg essential oil were *α*-pinene (20.16%), sabinene (14.45%), *β*-pinene (13.26%), and limonene (9.23%) [19]. Indeed, various factors, both biotic and abiotic, considerably impact the phytochemical profile of essential oils (e.g., agronomic factors such as climate, soil type, water/drought level, cultivation practices, insect and pathogen attacks, but also the time of harvesting, storage conditions, preliminary steps such as milling of plant material before essential oil extraction, or isolation method [6]).

### 2.2. LC-HRMS/MS Analysis of Nutmeg Residual Water, Spent, and Total Extracts

In the present study, a metabolite-profiling platform based on LC-HRMS/MS was used to phytochemically characterize three different nutmeg extracts. The residual water extract (NWE) was obtained by freeze-drying the water used for the hydrodistillation. The spent extract (NSE) was generated by processing the dried nutmeg biomass residue remaining at the end of the hydrodistillation. The total extract (NTE) was obtained from the raw dried powdered nutmeg seeds.

Fourteen compounds were tentatively identified and characterized based on their spectro-chromatographic data. Among them, four compounds (citric acid, quinic acid, hydroxybenzoic acid, catechin, and apigenin) were unequivocally identified by comparing their retention times and mass spectra with reference standards. The annotated compounds are presented in Table 2, along with their retention times, experimental and calculated *m*/*z*, mass error (ppm), molecular formula, MS/MS fragmentation ions, and distribution among extracts. The identified constituents were grouped into various phytochemical classes: organic/phenolic acids, flavonoids, lignans, and diarylnonanoids.

Citric acid (**1**), quinic acid (**2**), dihydroxybenzoic acid (**3**), and hydroxybenzoic acid (**5**) were included in the group of organic/phenolic acids. Next, three flavonoids were labeled in the nutmeg extracts. Catechin (**5**) presented the [M − H]^−^ ion at *m*/*z* 289.0722 (C_15_H_13_O_6_^−^) and diagnostic fragment ions at 245.0813 (C_14_H_13_O_4_^−^), 203.0701 (C_12_H_11_O_3_^−^), 151.0038 (C_8_H_7_O_3_^−^), and 125.0247 (C_6_H_5_O_3_^−^) [20]. The MS/MS spectrum of apigenin (**7**) with [M − H]^−^ at *m*/*z* 269.0452 showed characteristic ion fragment ions at *m*/*z* 241.0471 [M-CO-H]^−^, 227.0333 [M-C_2_H_2_O-H]^−^, and 201.0587 [M-C_3_O_2_-H]^−^ [21]. Compound **7** displayed the pseudo-molecular ion at *m*/*z* 271.0623, corresponding to a molecular formula of C_15_H_12_O_5_^−^. Its fragment ions at *m*/*z* 177.0182 (C_9_H_5_O_4_^−^), 151.0038 (C_8_H_7_O_3_^−^), and 119.0512 (C_8_H_7_O^−^) suggested the structure of naringenin [21].

**Figure 1:**
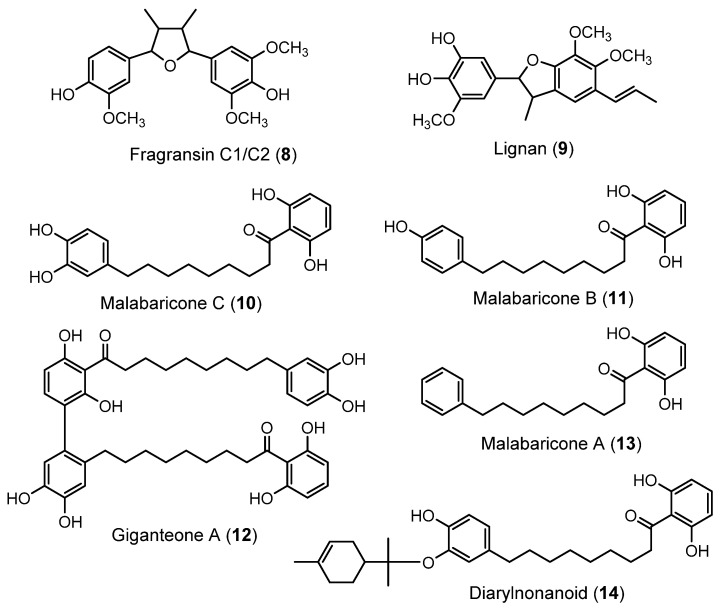
Chemical structures of lignans and diarylnonanoids tentatively identified by LC-HRMS/MS in nutmeg extracts.

Next, two lignans (**8** and **9**) and five diarylnonanoids (**10**–**14**) were tentatively labeled in the nutmeg samples (Figure 1). The first lignanic compound, peak **8** with [M − H]^−^ at *m*/*z* 373.1669 (C_21_H_25_O_6_^−^), was tentatively identified as fragransin C1/C2 [22]. In its MS/MS spectrum, the following elucidative fragments were noticed at *m*/*z* 355.1555 [M-H_2_O-H]^−^, 327.1199 [M-H_2_O-CO-H]^−^, 263.1300 [M-H_2_O-2×CH_2_O-CH_4_O-H]^−^, and 245.1182 [M-2×H_2_O-2×CH_2_O-CH_4_O-H]^−^. The fragment ion at *m*/*z* 249.1140 resulted from the loss of methoxyphenol from the molecule of fragransin C1/C2, whereas the fragment ion at *m*/*z* 123.0445 was assigned to [Methoxyphenol-H]^−^. Compound **9** ([M − H]^−^ at *m*/*z* 371.1491, C_21_H_23_O_6_^−^) was tentatively identified as 5-(6,7-dimethoxy-3-methyl-5-propenyl-2,3-dihydro-benzofuran-2-yl)-3-methoxy-benzene-1,2-diol, a lignan previously reported by Pandey et al. [23]. The cleavage of the furan ring gave the fragment ion at *m*/*z* 193.0860, whereas the removal of a CH_2_O group from this ion yielded the fragment at *m*/*z* 163.0752 [23].

Peaks **10**, **11**, and **13** were provisionally annotated as the diarylnonanoids malabaricone C, malabaricone B, and malabaricone A, respectively [23]. The fragment ions at *m*/*z* at 247.1325 for malabaricone C, 231.1387 for malabaricone A, and 215.1394 for malabaricone A resulted from the neutral loss of resorcinol. In addition, the three compounds shared the common fragment ions at *m*/*z* 135.0289 and 109.0304, corresponding to the formyl-resorcinol (C_7_H_3_O_3_^−^) and resorcinol (C_6_H_5_O_2_^−^) ions, respectively. As evidenced by its pseudo-molecular ion at *m*/*z* 713.3323, peak **12** indicated the putative structure of giganteone A (C_42_H_50_O_10_), formally obtained by the dimerization of malabaricone C. Due to the cleavage of the C–C bond, this compound produced the diagnostic fragment ion at *m*/*z* 355.1580 while the removal of a resorcinol group yielded the fragment ion at *m*/*z* 603.3014 [23]. Lastly, for compound **14** with the [M − H]^−^ ion at *m*/*z* 493.2989, the molecular formula C_31_H_42_O_5_ was suggested. The characteristic losses of resorcinol yielded the fragment ion at *m*/*z* 383.2594, while the loss of a *p*-menthene group (C_10_H_18_) generated the fragment ion at *m*/*z* 357.1668. This fragmentation pattern led to tentatively assigning the structure of peak **14** as 1-(2,6-dihydroxyphenyl)-9-[4-hydroxy-3-(p-menth-1-en-8-yloxy)phenyl]-1-nonanone, a compound previously reported in *M. fragrans* [24].

Concerning the inter-sample qualitative differences, it can be stated that the three nutmeg extracts had very similar profiles as all organic/phenolic acids, flavonoids, and diarylnonanoids were identified in all three samples. In contrast, the two lignans **8** and **9** were absent in the residual water extract. This could be linked with the low hydrophilicity of the two compounds, which makes them poorly extractible in water, or with their low stability in boiling aqueous solution.

**Table 2:** LC-HRMS/MS profile of nutmeg extracts.

No.	Compound	Class	T_R_ (min)	HRMS	Exp. (*m*/*z*)	Calcd. (*m*/*z*)	Δ (ppm)	MF	HRMS/MS (*m*/*z*)	Ref.	NWE	NSE	NTE
1	Citric acid *	Organic acid	2.7	[M − H]^−^	191.0199	191.0197	−0.91	C_6_H_8_O_7_	129.0198, 111.0100	[25]	×	×	×
2	Quinic acid *	Organic acid	5.9	[M − H]^−^	191.0566	191.0561	−2.54	C_7_H_12_O_6_	173.0431, 127.0395	[25]	×	×	×
3	Dihydroxybenzoic acid	Phenolic acid	13.1	[M − H]^−^	153.0198	153.0193	−3.04	C_7_H_6_O_4_	109.0284	[26]	×	×	×
4	Hydroxybenzoic acid *	Phenolic acid	15.3	[M − H]^−^	137.0239	137.0241	3.75	C_7_H_6_O_3_	119.0125, 109.0205	[26]	×	×	×
5	Catechin *	Flavonoid	20.9	[M − H]^−^	289.0722	289.0718	−1.51	C_15_H_14_O_6_	271.0454, 245.0813, 227.0736, 203.0701, 179.0367, 151.0396, 125.0230, 109.0218	[20]	×	×	×
6	Apigenin *	Flavonoid	30.6	[M − H]^−^	269.0452	269.0455	1.28	C_15_H_10_O_5_	241.0471, 227.0333, 201.0587, 185.0553, 169.0662, 133.0288	[21]	×	×	×
7	Naringenin	Flavonoid	33.8	[M − H]^−^	271.0623	271.0685	−4.05	C_15_H_12_O_5_	253.0523, 177.0182, 151.0038, 135.0265, 119.0512	[21]	×	×	×
8	Fragransin C1/C2	Lignan	39.2	[M − H]^−^	373.1669	373.1657	−3.31	C_21_H_26_O_6_	355.1555, 327.1199, 263.1300, 249.1140, 245.1182, 227.1215, 179.0398, 135.0259, 123.0445, 109.0287	[22]	-	×	×
9	5-(6,7-Dimethoxy-3-methyl-5-propenyl-2,3-dihydro-benzofuran-2-yl)-3-methoxy-benzene-1,2-diol	Lignan	43.1	[M − H]^−^	371.1491	371.1500	2.45	C_21_H_24_O_6_	327.1592, 261.1130, 217.1224, 193.0860, 178.0623,163.0393	[23]	-	×	×
10	Malabaricone C	Diarylnonanoid	48.6	[M − H]^−^	357.1704	357.1707	0.97	C_21_H_26_O_5_	313.1798, 289.1437, 247.1325, 135.0289, 109.0304	[27]	×	×	×
11	Malabaricone B	Diarylnonanoid	50.9	[M − H]^−^	341.1755	341.1758	0.10	C_21_H_26_O_4_	323.1649, 273.1498, 231.1387, 135.0085, 109.0297	[23]	×	×	×
12	Giganteone A	Diarylnonanoid	51.8	[M − H]^−^	713.3323	713.3331	1.15	C_42_H_50_O_10_	603.3014, 585.2901, 465.1956, 355.1580, 109.0296	[23]	×	×	×
13	Malabaricone A	Diarylnonanoid	53.6	[M − H]^−^	325.1803	325.1809	1.90	C_21_H_26_O_3_	307.1730, 257.1552, 215.1394, 145.0365, 135.0087, 109.0289	[23]	×	×	×
14	1-(2,6-Dihydroxyphenyl)-9-[4-hydroxy-3-(p-menth-1-en-8-yloxy)phenyl]-1-nonanone	Diarylnonanoid	54.7	[M − H]^−^	493.2989	493.2959	−3.95	C_31_H_42_O_5_	383.2594, 357.1668, 313.1813, 233.1184, 163.0420, 135.0160, 109.0289	[24]	×	×	×

MF, molecular formula; NSE, nutmeg spent extract; NTE, nutmeg total extract; NWE, nutmeg residual water extract; T_R_, retention time; * confirmed by standard; ×, present; -, absent; Δ, mass error.

### 2.3. Total Phenolic and Flavonoid Contents

Phenolic compounds are highly valued plant metabolites displaying various biological activities, including antimicrobial and anticancer properties [28]. In order to initiate the progress of phytochemical research, it is crucial to first assess these compounds qualitatively and quantitatively. In the first step, we detected the total amounts of phenolics and flavonoids using the Folin-Ciocalteu and AlCl_3_ methods, respectively. The results are shown in Table 3. NSE contained the highest total phenolics (63.31 mg GAE/g) and flavonoids (8.31 mg RE/g) contents, followed by NTE and NWE, respectively. Based on these results, the nutmeg spent material can be considered a valuable raw material with functional applications. This can be explained by removing carbohydrates and essential oil by distillation and, further, the hydroalcoholic solvent was more effective in extracting phenols or flavonoids. As mentioned in the literature, hydroalcoholic mixtures are preferential solvents for the extraction of phenolic compounds [29]. Further, we compared our results with literature data on crude plant material to reveal that the spent material is an important source of bioactive compounds. In a study by Rahman et al. [30], the total phenolic content in four nutmeg seed samples ranged from 0.174 to 1.891 mg GAE/100 g. Sulaiman and Ooi [31] documented a lower total phenolic content (46.3 mg GAE/g) in an 80% methanol extract of nutmeg seeds as compared to the value reported in our study. Pashapoor et al. [32] reported total phenolic and flavonoid levels in the petroleum ether of nutmeg seeds of 112.41 mg GAE/100g dry weight (DW) and 26.12 mg quercetin equivalents (QE)/100 g DW, respectively. The concentration of total phenolics in our samples was higher than those observed by Waman et al. [33] for various acetone:methanol 1:1 seed extracts, which ranged between 2.34–3.71 mg GAE/g. These variations can be assigned to geographical, climatic, and genetic factors.

### 2.4. Antioxidant Activity

Antioxidant compounds are important in managing oxidative stress associated with the progression of various diseases such as cancer, diabetes, and stroke [34,35]. Hence, the antioxidant capacity of a plant extract can offer valuable insights into its pharmaceutical potential. In the present study, the antioxidant abilities of nutmeg essential oil and extracts were determined by various in vitro assays, including free radical-scavenging (DPPH and ABTS), reducing power (FRAP and CUPRAC), metal-chelating, and total antioxidant (by phosphomolybdenum) assays. The results are presented in Table 4. The DPPH and ABTS assays detect the chain-breaking potential of plant extracts by measuring hydrogen transfer to free radicals. As shown in Table 4, the highest ability to scavenge radicals was demonstrated by NSE (DPPH: 49.18 mg TE/g; ABTS: 66.36 mg TE/g), but its activity was very close to that of NTE (*p* > 0.05). The lowest anti-radical scavenging capacity was exhibited by NWE (DPPH: 12.50 mg TE/g; ABTS: 21.04 mg TE/g). The NEO capacity to scavenge free radicals can be attributed to its main components (e.g., sabinene, myristicin, and *α*-pinene). These phytochemicals have previously been described as significant radical quenchers, supporting our findings [36,37,38]. Several authors reported on the free radical scavenging potentials of nutmeg seeds [39,40,41].

**Figure 2:**
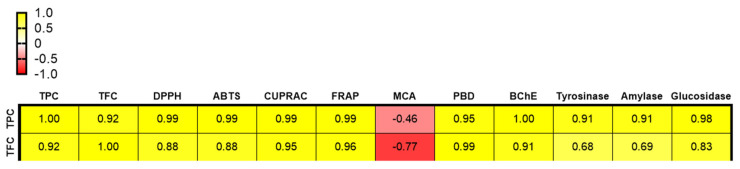
Pearson’s correlation between total phenolic (TPC)/total flavonoids contents (TFC) and antioxidant/enzyme inhibitory assays.

In addition to hydrogen transfer, the transfer of a single electron from antioxidants to metal ions, known as the reducing power, is also a significant antioxidant mechanism. To measure this ability, we conducted the FRAP and CUPRAC assays. In both tests, the highest reductive potential was displayed by NSE (FRAP: 105.28 mg TE/g and CUPRAC: 172.28 mg TE/g). NSE was followed by NTE and NEO in both FRAP and CUPRAC assays, respectively. In addition, the activity order was almost identical in the free radical-scavenging and power-reducing assays. Therefore, we concluded that these activities could be attributed to the same compounds. The correlation between phytocompounds (total phenolics and flavonoids) and biological activities is depicted in Figure 2 and clearly shows a strong interdependence between these parameters. Several researchers have reported findings consistent with ours, showing a linear correlation between the total amount of bioactive compounds and antioxidant properties [42,43,44]. In addition, compounds 8 and 9 (Table 2) were only detected in NSE and NTE, and these constituents could contribute to the overall capacity to quench free radicals and reduce metal ions.

The phosphomolybdenum method operates on the principle of transforming Mo (VI) to Mo (V) in an acidic environment. In this assay, the tested samples followed the order NEO > NSE > NTE > NEW in terms of bioactivity. Similarly, several authors reported that essential oils have higher activity compared to extracts in the phosphomolybdenum test [45,46]. Transition metals play a role in the Fenton and Haber-Weiss reactions and they contribute to the production of hydroxyl radical, the most deleterious among reactive oxygen species. In this context, metal chelation represents an important strategy in overcoming the production of hydroxyl radicals. Compared to other antioxidant assays, NTE exhibited the best metal chelating ability (25.16 mg EDTAE/g), followed by NWE (23.98 mg EDTAE/g) and NSE (15.14 mg EDTAE/g). Interestingly, the NEO showed no activity in the metal-chelating assay. As shown in Figure 2, no correlation has been found between the total bioactive compound content and the metal-chelating effects. These data suggest that the observed metal-chelating potential could be assigned to non-phenolic chelators such as polysaccharides and sulfides.

Taken together, our results prove that NSE possesses superior antioxidant activity compared to the other nutmeg extracts and highlight its potential use as a source of functional ingredients.

### 2.5. Enzyme Inhibition Activity

Currently, the prevalence of so-called “global health diseases” (e.g., Alzheimer’s, diabetes mellitus) has reached alarming levels and thus effective strategies to lower their social and economic burden are imperative. In the search for effective strategies, enzymes are considered a cornerstone in mitigating the physio-pathological picture of the above-mentioned diseases [47]. For example, acetylcholinesterase (AChE) hydrolyzes acetylcholine in synaptic cleavage. The inhibition of AChE can lead to an increase in acetylcholine levels and potentially alleviate the cognitive impairment associated with Alzheimer’s disease [48]. Similar observations have been made regarding the relationship between amylase/glucosidase and diabetes [43], as well as tyrosinase and hyperpigmentation [49]. With this in mind, several compounds have been designed and marketed as enzyme inhibitors. However, some studies have shown that most of these inhibitors cause deleterious side effects associated with their long-term use. Therefore, scientists are seeking alternative inhibitors to replace synthetic ones and plants are a pool of diverse chemical entities with high bioactive potential. Based on the preceding fact, we investigated the inhibitory effects of nutmeg samples derived from crude and by-product materials against cholinesterases, tyrosinase, amylase, and glucosidase. The obtained results are summarized in Table 5.

Although all samples showed inhibitory properties on butyrylcholinesterase (BChE), only NEO was active on AChE. The observed cholinesterase inhibitory effects of NEO could be due to the presence of monoterpenes (*α*-pinene, *β*-pinene, and sabinene, etc.) and alkenylbenzenes (myristicin). Previous studies have reported these compounds as cognitive enhancers [37,50,51]. As shown in Figure 2, the BChE inhibitory activity strongly correlated with the total bioactive compounds within the extracts. Consistent with our results, compounds such as malabaricone A and B isolated from nutmeg have been shown to exhibit significant cholinesterase (particularly BChE) inhibitory effects [52,53]. Similar to our data, Rastegari et al. [27] observed that different fractions of nutmeg were more active on BChE when compared to AChE. Concerning the tyrosinase inhibitory effects, NTE displayed the highest effect with 61.79 mg KAE/g, followed by NSE (47.74 mg KAE/g), NEO (46.40 mg KAE/g), and NWE (16.16 mg KAE/g). Literature reports showed that nutmeg extracts and isolated compounds possess significant anti-tyrosinase effects. For example, a previous study by Gao [54] mentioned malabaricone C as a potential tyrosinase inhibitor.

NTE showed the most effective inhibition on amylase and glucosidase, with values of 0.44 mmol ACAE for amylase and 1.87 mmol ACAE/g for glucosidase, respectively. However, NEO exhibited a stronger inhibitory effect on glucosidase compared to the other samples. This could be explained by the presence of several terpenes, including α/β-pinene, sabinene, and myristicin, which have been described as putative anti-diabetic agents [55,56,57]. In addition, malabaricones A, B, and C might contribute to these anti-amylase and anti-glucosidase activities, as reported by previous studies [58,59]. In conclusion, considering the significant enzyme-inhibiting properties, the spent material extract could be a valuable raw material for developing effective treatment strategies for the above-mentioned diseases.

### 2.6. Antimicrobial Activity

Nutmeg essential oil and extracts were previously reported to display antimicrobial activities against various human pathogens, including Gram-positive and Gram-negative bacteria, yeasts, and fungi [1,4]. The existing literature data prompted us to also assess the antimicrobial effects of nutmeg post-distillation by-products compared to nutmeg essential oil and total raw extract. The extracts were tested according to EUCAST guidelines against a panel of seventeen human pathogenic Gram-positive and Gram-negative bacteria and yeasts; the results of the antimicrobial screening are reported in Table 6.

The antimicrobial screening results were ranked using the criteria proposed by de Kuete [60], as follows: strong activity (MICs lower than 100 mg/L) and moderate-to-weak activity (MICs higher than 100 mg/L). As presented in Table 6, NSE showed strong activity against Gram-positive bacteria such as *Micrococcus luteus*, *Bacillus cereus*, and *Streptococcus pneumoniae* (MICs values of 62.5 mg/L). Furthermore, the MBC values revealed that NSE exhibited bactericidal effects against *Micrococcus luteus*. Concerning Gram-negative bacteria and yeasts, NSE displayed no significant antimicrobial effects. NEO, NEW, and NTE showed no significant antimicrobial activity against tested strains (MICs higher than 125 mg/L). Previously, Sulaiman et al. [31] reported MIC values of 50 mg/L for an 80% nutmeg methanol extract against *Staphylococcus aureus* and *B. cereus*. In addition, several solvent extracts (acetone, ethanol, methanol, aqueous, and butanol) derived from nutmeg seeds were active against *Staphylococcus aureus*, *Bacillus cereus*, and *Pseudomonas aeruginosa*, with MICs within the range 31.25–62.5 mg/L [61]. Regarding nutmeg essential oil, Piaru et al. [62] documented MIC values of 1000 mg/L against Gram-positive (*Staphylococcus aureus*, *Bacillus subtilis*) and Gram-negative (*Escherichia coli*, *Proteus mirabilis*, *Klebsiella pneumoniae*, *Salmonella thypi*, and *Pseudomonas aeruginosa*) bacteria. Overall, we can conclude that the spent plant material extract was more active than both nutmeg essential oil and raw extract and thus it can be regarded as a source of potential antibacterial agents.

## 3. Materials and Methods

### 3.1. Plant Material

Dried seeds of nutmeg (*Myristica fragrans* Houtt.) were purchased from the local market and identified by one of the authors (A.T.). A voucher specimen (MF/220920) was stored in the herbarium of the Department of Pharmacognosy-Phytotherapy, “Grigore T. Popa” University of Medicine and Pharmacy Iasi, Romania.

### 3.2. Extraction

#### 3.2.1. Isolation of Nutmeg Essential Oil

Nutmeg essential oil (NEO) was obtained by hydrodistillation using a Clevenger-type apparatus from ground nutmeg seeds (100 g plant material, 1000 mL distilled water, 3 h extraction time). The isolation procedure was performed in duplicate and the derived essential oil was kept in dark glass tubes at 4 °C until subsequent analysis. The obtained NEO yielded 7.05 ± 0.35 mL%.

#### 3.2.2. Obtaining Nutmeg Residual Water, Spent and Total Extracts

After hydrodistillation, the remaining water in the distillation flask was filtered, then 25 mL was freeze-dried, thus obtaining the residual water extract (NWE). The solid plant residue (spent material) was first dried (40 °C, for 48 h), then 5 g were subjected to extraction using methanol/water 75/25 (*v*/*v*) (50 mL, in three rounds of 30 min each, by ultra-sonication). The solvent was evaporated under vacuum and the obtained extract (NSE) was stored at −20 °C for further analysis. As a comparison, crude nutmeg seeds (5 g) were ground and extracted using methanol/water 75/25 (*v*/*v*) following the methodology mentioned above. The resulting extract (total extract, NTE) was dried under vacuum and kept at −20 °C until subsequent analysis. Each experimental procedure was performed in duplicate. The extraction yields for NWE, NSE, and NTE were 22.58 ± 3.09%, 5.15 ± 1.00%, and 8.58 ± 0.93%, respectively.

### 3.3. Phytochemical Screening

The total phenolic (TPC) and flavonoid contents (TFC) were assessed by Folin–Ciocalteu and aluminium chloride methods, respectively [25]. The results were expressed as gallic acid equivalents (mg GAE/g extract) and rutin equivalents (mg RE/g extract) for TPC and TFC, respectively. The GC-MS analysis of NEO and LC-HRMS/MS analysis of NWE, NSE, and NTE were undertaken following the methodologies extensively detailed in Luca et al. [26].

### 3.4. Antioxidant and Enzyme Inhibition Assays

DPPH and ABTS radical-scavenging, ferric- and cupric-reducing antioxidant power, metal-chelating capacity, total antioxidant ability (phosphomolybdenum method), and inhibition of cholinesterases (acetylcholinesterase, butyrylcholinesterase), tyrosinase, amylase, and glucosidase assays were assessed following previously reported methods [25] (see Supplemental Material). Each nutmeg sample was tested in triplicate.

### 3.5. Antimicrobial Assays

The antimicrobial assays were undertaken by the microdilution method following the European Committee on Antimicrobial Susceptibility Testing guidelines [63]. MH broth and MH broth with 7% lysed horse blood were employed to grow non-fastidious bacteria, whereas MH broth with 2% glucose was used to grow yeasts. All tests were performed in triplicate. The minimum inhibitory concentration (MIC) and minimum bactericidal concentration (MBC) of the nutmeg samples were assessed for Gram-positive bacteria (*Bacillus cereus* ATCC 10876, *Enterococcus faecalis* ATCC 29212, *Micrococcus luteus* ATCC 10240, *Staphylococcus aureus* ATCC 25923, *S. aureus* ATCC BAA-1707, *S. epidermidis* ATCC 12228, *Streptococcus pneumoniae* ATCC 49619, *S. pyogenes* ATCC 19615, and *S. mutans* ATCC 25175); Gram-negative bacteria (*Escherichia coli* ATCC 25922, *Klebsiella pneumoniae* ATCC 13883, *Proteus mirabilis* ATCC 12453, *Pseudomonas aeruginosa* ATCC 9027, and *Salmonella* Typhimurium ATCC 14028); and yeasts (*Candida albicans* ATCC 2091, *C. glabrata* ATCC 90030, and *C. parapsilosis* ATCC 22019).

## 4. Conclusions

Our study assessed for the first time the phytochemical (GC-MS, LC-HRMS/MS) and biological (antioxidant, enzyme inhibitory, antimicrobial) profiles of nutmeg crude materials (essential oil and total extract) in comparison to post-distillation by-products (residual water and spent material extract). Altogether, we can conclude that nutmeg spent material is an important source of bioactive compounds (e.g., organic acids, flavonoids, phenolic acids, lignans, and diarylnonanoids). Moreover, the observed antioxidant, enzyme inhibitory, and antimicrobial activities of the oil-exhausted biomass bear evidence for its health-promoting potential and refer it as a suitable material for the food, pharmaceutical, and cosmeceutical industries. In addition, our data support the valorization of large amounts of nutmeg post-distillation by-products, with significant ecological and economic impact.

## Figures and Tables

**Table 1:** GC-MS profile of nutmeg essential oil.

No.	Compound	LRI ^a^	(%) ^b^
1	3-Thujene	927	2.33 ± 0.01
2	*α*-pinene	935	15.81 ± 0.26
3	Camphene	951	0.28 ± 0.01
4	Sabinene	974	21.71 ± 0.52
5	*β*-Pinene	979	12.70 ± 0.52
6	*β*-Myrcene *	989	2.04 ± 0.04
7	α-Phellandrene	1006	0.47 ± 0.01
8	3-Carene	1009	0.77 ± 0.01
9	α-Terpinene	1018	1.42 ± 0.03
10	p-Cymene	1025	0.76 ± 0.02
11	Limonene *	1030	5.86 ± 0.09
12	γ-Terpinene	1060	2.24 ± 0.04
13	cis-α-Terpineol	1072	0.47 ± 0.01
14	α-Terpinolene	1086	0.99 ± 0.02
15	*p*-Cymenene	1090	0.04 ± 0.00
16	Linalool *	1098	0.33 ± 0.01
17	*trans*-5-Caranol	1101	0.44 ± 0.01
18	*cis*-*p*-Menth-2-en-1-ol	1126	0.24 ± 0.01
19	*trans*-*p*-Menth-2-en-1-ol	1144	0.14 ± 0.01
20	Terpinen-4-ol	1183	3.73 ± 0.08
21	*p*-Cymen-8-ol	1188	0.04 ± 0.00
22	*trans-α*-Terpineol	1196	0.61 ± 0.02
23	Bornyl acetate	1285	0.14 ± 0.01
24	Safrole	1291	2.55 ± 0.06
25	Isopulegol acetate	1296	0.13 ± 0.00
26	Myrtanyl acetate	1343	0.26 ± 0.01
27	Eugenol	1350	0.44 ± 0.02
28	Geraniol acetate	1374	0.16 ± 0.01
29	Copaene	1380	0.74 ± 0.03
30	*α*-Cubenene	1391	0.06 ± 0.00
31	Methyleugenol	1398	5.13 ± 0.12
32	Caryophyllene *	1427	0.23 ± 0.01
33	(Z)-*α*-Bergamotene	1436	0.15 ± 0.01
34	Isoeugenol	1449	0.53 ± 0.02
35	Humulene *	1464	0.04 ± 0.01
36	Germacrene D	1488	0.22 ± 0.01
37	Methylisoeugenol	1494	1.20 ± 0.03
38	*γ*-Elemene	1503	0.12 ± 0.01
39	*β*-Bisabolene	1510	0.10 ± 0.00
40	Myristicin	1524	13.39 ± 0.10
41	*α*-Bisabolene	1544	0.65 ± 0.02
42	Methoxyeugenol	1594	0.06 ± 0.02
43	Guaiol	1603	0.02 ± 0.02
	*Hydrocarbon monoterpenes*		68.50 ± 0.60
	*Oxygenated monoterpenes*		6.41 ± 0.13
	*Hydrocarbon sesquiterpenes*		1.51 ± 0.04
	*Oxygenated sesquiterpenes*		0.02 ± 0.02
	*Aromatic compounds*		23.31 ± 0.31
	Total identified		99.79 ± 0.09

^a^ Retention index on ZB-5MS column; ^b^ expressed as the mean percentage area ± standard deviation; * Confirmed by authentic standard.

**Table 3:** Total phenolic and flavonoid contents of nutmeg extracts.

Sample	Total Phenolic Content (mg GAE/g)	Total Flavonoid Content (mg RE/g)
NWE	10.02 ± 0.02 ^c^	2.12 ± 0.19 ^c^
NSE	63.31 ± 0.72 ^a^	8.31 ± 0.06 ^a^
NTE	57.42 ± 3.90 ^b^	5.33 ± 0.06 ^b^

Results are shown as the mean ± standard deviation of three replicates; significant differences in the investigated samples (*p* < 0.05) are indicated by different letters within columns. NSE, nutmeg spent extract; NTE, nutmeg total extract; NWE, nutmeg residual water extract; GAE, gallic acid equivalents; RE, rutin equivalents.

**Table 4:** The antioxidant activity of nutmeg essential oil and extracts.

Sample	DPPH (mg TE/g)	ABTS (mg TE/g)	CUPRAC (mg TE/g)	FRAP (mg TE/g)	Metal Chelating (mg EDTAE/g)	Phosphomolybdenum (mmol TE/g)
NEO	28.61 ± 0.35 ^b^	60.20 ± 0.61 ^b^	113.74 ± 3.09 ^c^	105.28 ± 1.93 ^b^	n.a.	57.99 ± 0.19 ^a^
NWE	12.50 ± 0.56 ^c^	21.04 ± 0.41 ^c^	22.27 ± 0.29 ^d^	16.27 ± 0.11 ^d^	23.98 ± 0.31 ^a^	0.36 ± 0.01 ^d^
NSE	49.18 ± 0.13 ^a^	66.36 ± 0.04 ^a^	172.28 ± 2.66 ^a^	108.11 ± 3.18 ^a^	15.14 ± 1.48 ^b^	4.00 ± 0.20 ^b^
NTE	49.12 ± 0.17 ^a^	66.15 ± 0.17 ^a^	144.78 ± 4.36 ^b^	86.52 ± 0.94 ^c^	25.16 ± 1.92 ^a^	2.61 ± 0.05 ^c^

Results are shown as the mean ± standard deviation of three replicates; significant differences in the investigated samples (*p* < 0.05) are indicated by different letters within columns. EDTAE, EDTA equivalents; n.a., not active; NEO, nutmeg essential oil; NSE, nutmeg spent extract; NTE, nutmeg total extract; NWE, nutmeg residual water extract; TE, Trolox equivalents.

**Table 5:** Enzyme inhibitory activity of nutmeg essential oil and extracts.

Sample	Acetylcholinesterase (mg GALAE/g)	Butyrylcholinesterase (mg GALAE/g)	Tyrosinase (mg KAE/g)	Amylase (mmol ACAE/g)	Glucosidase (mmol ACAE/g)
NEO	4.04 ± 0.14	4.21 ± 0.02 ^b^	46.40 ± 2.39 ^b^	0.33 ± 0.01 ^b^	1.90 ± 0.07 ^a^
NWE	n.a.	2.81 ± 0.03 ^c^	16.16 ± 0.42 ^c^	0.16 ± 0.02 ^c^	n.a.
NSE	n.a.	4.78 ± 0.03 ^a^	47.74 ± 4.58 ^b^	0.35 ± 0.02 ^b^	1.69 ± 0.08 ^b^
NTE	n.a.	4.61 ± 0.05 ^a^	61.79 ± 2.39 ^a^	0.44 ± 0.00 ^a^	1.87 ± 0.01 ^a^

Results are shown as the mean ± standard deviation of three replicates; significant differences in the investigated samples (*p* < 0.05) are indicated by different letters within columns. ACAE, acarbose equivalents; GALAE, galanthamine equivalents; KAE, kojic acid equivalents; n.a., not active; NEO, nutmeg essential oil; NSE, nutmeg spent extract; NTE, nutmeg total extract; NWE, nutmeg residual water extract.

**Table 6:** Antimicrobial properties of nutmeg essential oil and extracts.

Microorganism	NEO		NWE		NSE		NTE		Control
	MIC (mg/L)	MBC (mg/L)	MIC (mg/L)	MBC (mg/L)	MIC (mg/L)	MBC (mg/L)	MIC (mg/L)	MBC (mg/L)	MIC (mg/L)
**Gram-positive bacteria**									Vancomycin
*Bacillus cereus* ATCC 10876	2000	>2000	>2000	n.d.	62.5	2000	250	>2000	0.98
*Enterococcus faecalis* ATCC 29212	2000	>2000	>2000	n.d.	500	>2000	1000	>2000	1.95
*Micrococcus luteus* ATCC 10240	2000	>2000	>2000	n.d.	62.5	125	125	250	0.12
*Staphylococcus aureus* ATCC 25923	>2000	>2000	>2000	n.d.	125	250	250	250	0.98
*Staphylococcus aureus* ATCC BAA-1707 *	>2000	>2000	>2000	n.d.	125	250	250	500	0.98
*Staphylococcus epidermidis* ATCC 12228	>2000	>2000	>2000	n.d.	500	>2000	1000	>2000	0.98
*Streptococcus pneumoniae* ATCC 49619	1000	2000	>2000	n.d.	62.5	1000	250	1000	0.24
*Streptococcus pyogenes* ATCC 19615	2000	>2000	>2000	n.d.	250	>2000	1000	>2000	0.24
*Streptococcus mutans* ATCC 25175	2000	>2000	>2000	n.d.	1000	>2000	>2000	>2000	0.98
**Gram-negative bacteria**									Ciprofloxacin
*Escherichia coli* ATCC 25922	>2000	n.d.	>2000	n.d.	2000	n.d.	>2000	n.d.	0.015
*Klebsiella pneumoniae* ATCC 13883	>2000	n.d.	>2000	n.d.	2000	n.d.	>2000	n.d.	0.122
*Proteus mirabilis* ATCC 12453	>2000	n.d.	>2000	n.d.	2000	n.d.	>2000	n.d.	0.030
*Pseudomonas aeruginosa* ATCC 9027	>2000	n.d.	>2000	n.d.	2000	n.d.	2000	n.d.	0.488
*Salmonella* Typhimurium ATCC 14028	>2000	n.d.	>2000	n.d.	2000	n.d.	>2000	n.d.	0.061
**Yeasts**									Nystatin
*Candida albicans* ATCC 102231	2000	>2000	>2000	>2000	1000	>2000	2000	>2000	0.24
*Candida glabrata* ATCC 2091	1000	>2000	>2000	>2000	2000	>2000	2000	>2000	0.48
*Candida parapsilosis* ATCC 22019	250	2000	2000	>2000	250	>2000	500	>2000	0.24

MBC, minimum bactericidal concentration; MIC, minimum inhibitory concentration; n.d., not determined; NEO, nutmeg essential oil; NSE, nutmeg spent extract; NTE, nutmeg total extract; NWE, nutmeg residual water extract; * methicillin-resistant *Staphylococcus aureus* (MRSA) strain.

## Supplementary Materials

The following supporting information can be downloaded at: https://www.mdpi.com/article/10.3390/plants12091741/s1.
