# Supplementary material for: Essential Oils and Sustainability: In Vitro Bioactivity Screening of Myristica fragrans Houtt. Post-Distillation By-Products
Source: Plants (Basel). 2023 Apr 23;12(9):1741. doi: 10.3390/plants12091741 (PMC10181112; doi:10.3390/plants12091741)
Supplement: Supplementary file 1 [file plants-12-01741-s001.zip › plants-2354697-supplementary.pdf]

Antioxidant and Enzyme Inhibitory Assays

DPPH (1,1-diphenyl-2-picrylhydrazyl) radical scavenging assay

Sample solution (50 μL) was added to 150 μL of a 0.004% methanol solution of DPPH. The sample absorbance was read at 517 nm in a 96-well microplate after 30 min of incubation (at room temperature, in the dark). DPPH radical scavenging activity was expressed as milligrams of Trolox equivalents (mg TE/g extract).

ABTS (2,2′-azino-bis(3-ethylbenzothiazoline) 6-sulfonic acid) radical scavenging assay

Briefly, ABTS+ was produced directly by reacting 7 mM ABTS solution with 2.45 mM potassium persulfate and allowing the mixture to stand for 12–16 min (in the dark, at room temperature). Prior to the assay, ABTS solution was diluted in methanol to an absorbance of 0.700 ± 0.02 at 734 nm. Sample solution (25 μL) was added to ABTS solution (200 μL) and mixed. The sample absorbance was read at 734 nm in a 96-well microplate after 30 min of incubation at room temperature. The ABTS radical scavenging activity was expressed as milligrams of Trolox equivalents (mg TE/g extract).

CUPRAC (cupric ion reducing activity) activity assay

Sample solution (25 μL) was added to premixed reaction mixture (200 μL) containing $CuCl_2$ (1 mL, 10 mM), neocuproine (1 mL, 7.5 mM) and NH_4Ac buffer (1 mL, 1 M, pH 7.0). Similarly, a blank was prepared by adding sample solution (25 μL) to a premixed reaction mixture (200 μL) without $CuCl_2$. Then, the sample and blank absorbances were read at 450 nm in a 96-well microplate after 30 min of incubation at room temperature. The absorbance of the blank was subtracted from that of the sample. CUPRAC activity was expressed as milligrams of Trolox equivalents (mg TE/g extract).

FRAP (ferric reducing antioxidant power) activity assay

Sample solution (25 μL) was added to premixed FRAP reagent (200 μL) containing acetate buffer (0.3 M, pH 3.6), 2,4,6-tris(2-pyridyl)-S-triazine (TPTZ) (10 mM) in 40 mM HCl and ferric chloride (20 mM) in a ratio of 10:1:1 (*v*/*v*/*v*). Then, the sample absorbance was read at 593 nm in a 96-well microplate after 30 min of incubation at room temperature. FRAP activity was expressed as milligrams of Trolox equivalents (mg TE/g extract).

Phosphomolybdenum assay

Sample solution (100 μL) was combined with 3 mL of reagent solution (0.6 M sulfuric acid, 28 mM sodium phosphate and 4 mM ammonium molybdate). The sample absorbance was read at 695 nm in a 96-well microplate after 90 min of incubation at 95 °C. The total antioxidant capacity was expressed as millimoles of Trolox equivalents (mmol TE/g extract).

Metal chelating activity assay

Briefly, sample solution (100 μL) was added to $FeCl_2$ solution (50 μL, 2 mM). The reaction was initiated by the addition of 5 mM ferrozine (100 μL). Similarly, a blank was prepared by adding sample solution (100 μL) to $FeCl_2$ solution (50 μL, 2 mM) and water (100 μL) without ferrozine. Then, the sample and blank absorbances were read at 562 nm in a 96-well microplate after 10 min of incubation at room temperature. The absorbance of the blank was subtracted from that of the sample. The metal chelating activity was expressed as milligrams of EDTA (disodium edetate) equivalents (mg EDTAE/g extract).

Cholinesterase (ChE) inhibitory activity assay

Sample solution (100 μL) was mixed with DTNB (5,5-dithio-bis(2-nitrobenzoic) acid) (125 μL) and AChE (acetylcholinesterase (Electric ell acetylcholinesterase, Type-VI-S, EC 3.1.1.7, Sigma)), or BChE (butyrylcholinesterase (horse serum butyrylcholinesterase, EC 3.1.1.8, Sigma)) solution (25 μL) in Tris–HCl buffer (pH 8.0) in a 96-well microplate and incubated for 15 min at 25 °C. The reaction was then initiated with the addition of acetylthiocholine iodide or butyrylthiocholine chloride (25 μL). Similarly, a blank was prepared by adding sample solution to all reaction reagents without enzyme (AChE or BChE) solution. The sample and blank absorbances were read at 405 nm after 10 min of incubation at 25 °C. The absorbance of the blank was subtracted from that of the sample and the cholinesterase inhibitory activity was expressed as galanthamine equivalents (mg GALAE/g extract).

Tyrosinase inhibitory activity assay

Sample solution (50 μL) was mixed with tyrosinase solution (40 μL, Sigma) and phosphate buffer (100 μL, pH 6.8) in a 96-well microplate and incubated for 15 min at 25 °C. The reaction was then initiated with the addition of L-DOPA (40 μL, Sigma). Similarly, a blank was prepared by adding sample solution to all reaction reagents without enzyme (tyrosinase) solution. The sample and blank absorbances were read at 492 nm after 10 min of incubation at 25 °C. The absorbance of the blank was subtracted from that of the sample and the tyrosinase inhibitory activity was expressed as kojic acid equivalents (mg KAE/g extract).

α-amylase inhibitory activity assay

Sample solution (50 μL) was mixed with α-amylase solution (ex-porcine pancreas, EC 3.2.1.1, Sigma) (50 μL) in phosphate buffer (pH 6.9 with 6 mM sodium chloride) in a 96-well microplate and incubated for 10 min at 37 °C. After pre-incubation, the reaction was initiated with the addition of starch solution (50 μL, 0.05%). Similarly, a blank was prepared by adding sample solution to all reaction reagents without enzyme (α-amylase) solution. The reaction mixture was incubated 10 min at 37 °C. The reaction was then stopped with the addition of HCl (25 μL, 1 M). This was followed by addition of the iodine-potassium iodide solution (100 μL). The sample and blank absorbances were read at 630 nm. The absorbance of the blank was subtracted from that of the sample and the α-amylase inhibitory activity was expressed as acarbose equivalents (mmol ACE/g extract).

α-glucosidase inhibitory activity assay

Sample solution (50 μL) was mixed with α-glucosidase solution (from *Saccharomyces cerevisiae*, EC 3.2.1.20, Sigma) (50 μL) in phosphate buffer (pH 6.8) and PNPG (4-N-trophenyl-α-D-glucopyranoside, Sigma) (50 μL) in a 96-well microplate and incubated for 15 min at 37 °C. Similarly, a blank was prepared by adding sample solution to all reaction reagents without enzyme (α-glucosidase) solution. The reaction was then stopped with the addition of sodium carbonate (50 μL, 0.2 M). The sample and blank absorbances were read at 400 nm. The absorbance of the blank was subtracted from that of the sample and the α-glucosidase inhibitory activity was expressed as acarbose equivalents (mmol ACE/g extract).
